# Direct laser writing of black metals for tuneable plasmonic nanoparticles: experimental and computational insights

**DOI:** 10.1039/d6ra00590j

**Published:** 2026-05-27

**Authors:** Taavi Repän, Martin Hruška, Jaroslav Otta, Tereza Hodná, Přemysl Fitl, Michal Novotný, Martin Vrňata, Raivo Jaaniso

**Affiliations:** a Institute of Physics, University of Tartu Tartu 50411 Estonia taavi.repan@ut.ee; b Department of Physics and Measurements, University of Chemistry and Technology Prague Technická 5 166 28 Prague 6 Czech Republic; c Institute of Physics, Czech Academy of Sciences Na Slovance 2 182 21 Prague 8 Czech Republic

## Abstract

Plasmonic nanoparticles, recognized for their light-absorbing qualities, show significant potential in many optical applications including nanophotonics and sensors. However, their widespread use is often limited by the high cost associated with manufacturing processes. In this study, we introduce an accessible and cost-effective approach to produce plasmonic nanoparticles. We employ a straightforward direct laser writing method, utilizing a common 405 nm continuous-wave laser diode, on nanostructured black metal films that can readily melt to produce spherical nanoparticles. By adjusting the laser power, we can control the size of these particles and arrange them in patterns on the black metal films. Subsequently, the optical properties of the nanoparticles are characterized and the experimental data are related with those obtained by computational simulations. The analysis indicates that two separate particle populations are formed during laser writing. The analysis attributes the observed absorption peak to plasmonic resonances in the particles population with smaller diameter. The formation of the particles is controlled by the writing laser power, affecting size and shape distribution of the particles, and subsequently—plasmonic resonances. This approach holds the potential to enable the economical production of plasmonic nanoparticles, which could have broad applications.

## Abstract 2

A direct laser writing method transforms porous black gold into discrete plasmonic nanoparticles. Adjusting laser power tunes nanoparticle size and shape distributions, and thus the plasmonic resonances.

## Graphical abstract

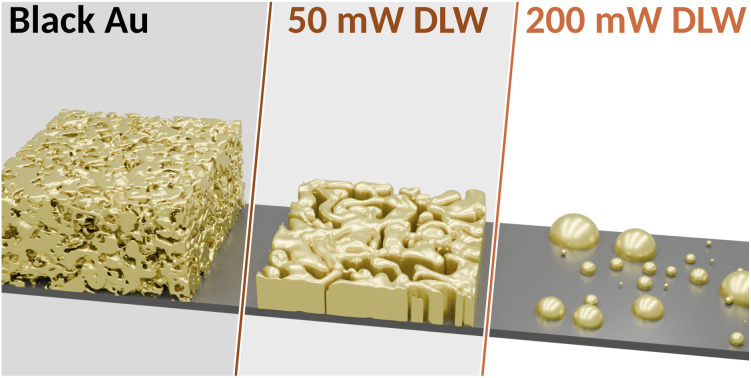

## 1 Introduction

Thin film nanomaterials, composed of nanoparticles or nanostructures, often exhibit physical properties markedly distinct from their bulk counterparts. These properties are particularly interesting because they open pathways to novel optical, electronic, and catalytic functionalities.^1,2^ One promising class of materials with such properties are black metals (BMs). These are highly nanostructured metallic layers that have historically attracted broad attention due to their strong absorption in the visible and near-infrared regions. First described by Pfund,^3^ black metals prepared by thermal evaporation at elevated inert gas pressures reached the height of their prominence in the 1950s–1970s, when their exceptional light-trapping behaviour was extensively investigated.^4–9^ Black gold (BAu) is one representative BM and can be prepared by thermal evaporation of Au in an inert gas atmosphere at elevated pressure. Gas-phase collisions reduce the kinetic energy of the arriving species and suppress surface diffusion, yielding a highly porous “cauliflower-like” morphology.^10,11^ In contrast, Au films evaporated under high vacuum are comparatively compact and reflective, and therefore lack the strong broadband light-trapping response characteristic of BAu.^10^ The early work on nanostructured metallic layers was subsequently extended by numerous groups who broadened the category of black metals—and black coatings in general—through a variety of alternative fabrication approaches. These include femtosecond laser structuring,^12^ galvanic replacement *via* dealloying,^13^ metal deposition onto pre-patterned substrates such as etched silicon,^14^ and physical vapour deposition techniques including magnetron sputtering.^15,16^ As a result, the field of black coatings has grown considerably. However, when such coatings are compared, careful attention must be paid to the specific manufacturing process, as the resulting morphology and properties are highly process-dependent.

In recent years, evaporated black metals have been undergoing a certain renaissance. This renewed interest has been particularly evident in the field of gas sensing, where the high specific surface area of black metal layers has been exploited to enhance the receptor function of the sorbent in quartz crystal microbalance sensors, resulting in an increased sensitivity and lowered detection limit.^17^ Promising results were obtained also for chemiresistors with sensitive layers based on BMs.^10,18^ Pulsed UV laser treatment was applied to vary black gold optical properties by micro and nanostructure modification.^19^

In the present study, we demonstrate that direct laser writing (DLW) with a continuous-wave laser can be employed to selectively restructure and pattern black metal surfaces, resulting in spatially defined plasmonic nanoparticles. Although the process requires preparation of black metal layers as a preliminary step, it offers a straightforward and lithography-free route to nanoparticle patterning, distinguishing it from more conventional nanofabrication approaches that typically rely on expensive or complex instrumentation. Compared to cleanroom lithography and laser surface modification approaches that often rely on femtosecond systems, our workflow combines scalable thin-film deposition with mask-free patterning using a low-power continuous-wave diode and a motorised translation stage.^15^ Reproducibility is supported by instrument-defined process parameters (incident power and scan speed) together with controlled BAu deposition conditions.

The properties of plasmonic nanoparticles (NPs) are highly sensitive to their local dielectric environment and particle shape, which strongly influence their localised surface plasmon resonance (LSPR). This sensitivity makes 2D NP structures particularly attractive for applications in nanophotonics,^20–24^ bio-devices,^25,26^ sensors, single-molecule detection,^27^ and other analytical approaches.^28^ Their diffractive properties also enable their use in light trapping for solar cells^29^ and document security.^30^ Therefore, controlling NP size, shape, arrangement, and environment during growth is critical to tuning their optical response.^31^ In dense nanoparticle ensembles, near-field coupling can generate collective resonances and electromagnetic hot spots that influence both near-field enhancement, far-field spectra^32^ and hybrid plasmonic functionalities.^33^ In nanoparticle-on-metal geometries, additional coupling to propagating modes in the underlying film can further modify the optical response.^34^

Conventionally, plasmonic nanoparticle structures have been fabricated using ‘top-down’ approaches, where subtractive techniques are applied to sculpt uniform materials into nanoscale architectures. These methods, such as focused ion beam milling,^35,36^ electron beam lithography,^37^ nanoimprint lithography,^38^ and plasmon-induced lithography^39,40^—enable precise control over particle shape and spatial arrangement, often achieving resolution at the tens-of-nanometre scale. However, their high cost, limited throughput, and time-consuming workflows hinder their use in scalable applications that require large-area patterning^41^ In response, various ‘bottom-up’ strategies have been explored, including template-assisted growth,^42^ thermal annealing,^43^ pattern transfer.^44^ Although these additive techniques are typically more cost-effective and scalable, they often suffer from limited precision and reproducibility.^45^ Among them, laser-based methods, particularly DLW, have emerged as highly promising for controlling the ‘bottom-up’ strategies.^46^

Herein, we demonstrate a simple and accessible approach to fabricate plasmonic nanoparticles using direct laser writing (DLW) on nanostructured black gold (BAu) substrates. By selectively irradiating the BAu surface with a 405-nm continuous-wave laser diode (200 mW maximum power), we induce localised photothermal effects that transform the nanostructured gold layer. This process leads to the formation of discrete nanoparticles that exhibit localised surface plasmon resonance (LSPR). Our findings reveal that the DLW technique can reliably pattern porous black gold structures into plasmonic nanoparticle films with controllable particle formation. Moreover, through detailed numerical modelling, we link the tuneable optical response to variations in particle size and shape distributions, putting into context the results of numerical simulations, optical absorbance measurements, and scanning electron microscopy (SEM) image analysis.

## 2 Results and discussion

### 2.1 Direct laser writing: fabricating plasmonic NPs

We first examine a method to modify the optical properties of black gold layers through laser-induced melting. We used a continuous-wave laser with an XYZ micropositioning stage for laser direct writing (DLW). This setup offers different scan speeds to control the overall delivered energy density, making it feasible to melt down the nanostructure clusters in a manner similar to temperature-induced melting. This process offers flexibility in controlling the transformation of black gold films into LSPR nanoparticles.

The energy density provided, which causes the melting of nanostructured gold to form a spherical nanoparticle, can be controlled either by the laser scan speed when fixing the laser power, or by the laser power when fixing the scan speed, thus making the process of DLW highly controllable. For example, for 50 mW laser power, there is an evident threshold around the scan speed of 0.1 mm s^−1^, when the melted layer starts to form a higher number of spherical NPs, as evidenced by the SEM and AFM images [see Fig. S1.1 in the (SI)].

**Fig. 1:**
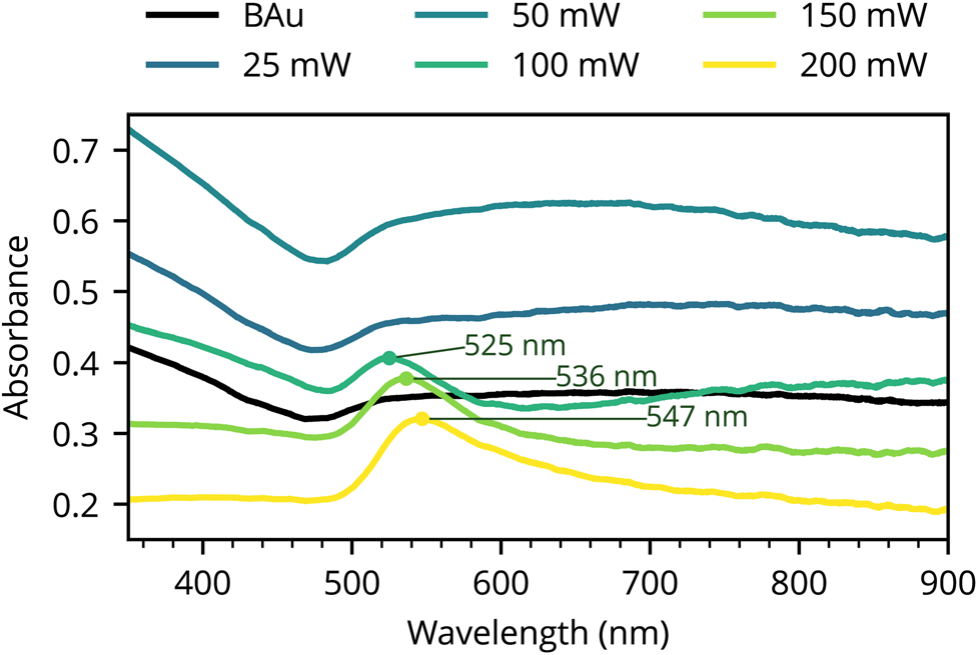
Absorbance spectra of nanoparticles fabricated by DLW.

In the following results, we have fixed the writing speed to 10 mm s^−1^ and varied the DLW laser power up to 200 mW. The plasmonic behaviour is evident in the absorbance spectra illustrated in Fig. 1. In particular, samples obtained using the three highest power levels (100 mW and above) show absorption peaks around 525–547 nm. DLW-treated samples were re-measured after one year of ambient storage, and only a negligable changes in LSPR peak positions has been observed (Fig. S7.1 in the SI). We attribute these variations to surface contamination during ambient exposure (*e.g.*, adsorption of airborne carbonaceous species), which is commonly observed for highly porous metallic films and has been reported previously.^10^

**Fig. 2:**
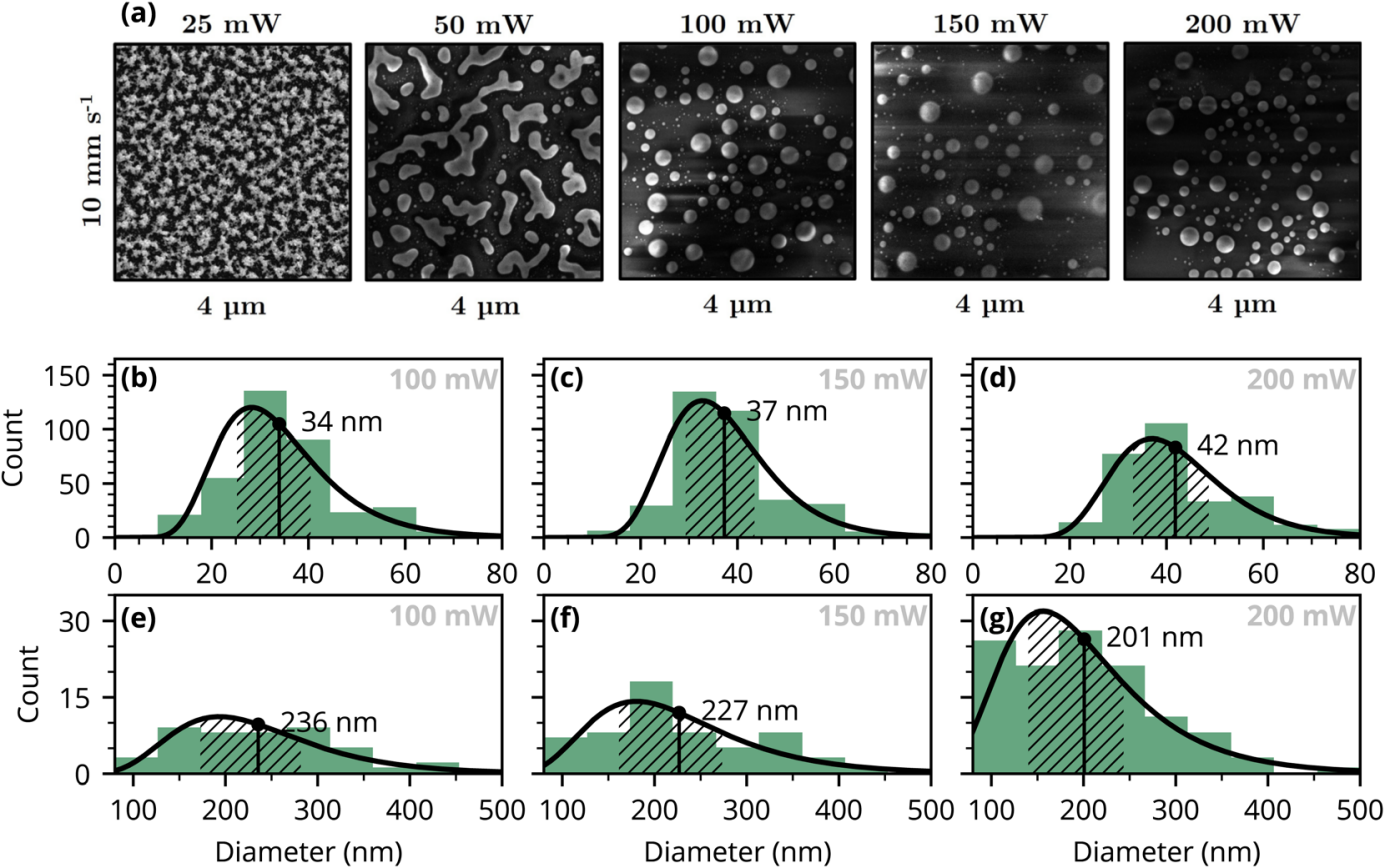
(a) SEM images illustrating the formation of NPs, using different laser powers 50–200 mW with a constant scan speed of 10 mm s^−1^. (b–g) The assumed log-normal distributions for particle sizes for the smaller (b–d) and the larger particle sets (e–g).

SEM images of the formed structures for various laser powers are shown in Fig. 2(a). The particle analysis made on the SEM images [see Fig. 2(b–g)] reveal two distinct sets of particles: the first set comprises smaller particles with diameters below 80 nm, while the larger set consists of particles with diameters mostly between 150 to 300 nm. The formation of two particle populations follows from the two pathways in the particle formation—the black gold is a porous network with at least two characteristic length scales (thin branches and thicker nodal aggregates).^10,47^ Local melting and coalescence of thicker nodes drive the formation of smaller number of larger droplets, while the finer branches undergo rounding and breakup, producing a population of small particles. For the latter pathway the particle formation is constrained by the local gold volume in the initial ligament.

Interestingly, increasing laser power results in a slight growth in the mean diameter of these smaller nanoparticles: from 34 to 42 nm (as the power goes from 100 to 200 mW). Conversely, the second set of larger nanoparticles demonstrates a reduction in diameter with higher laser power: from 236 to 201 nm. It should be noted, however, that the observed decrease in diameter do not necessarily imply a decrease in the particle volume, due to changes in the particle shape (contact angle). The ratio (*Θ*), representing the number of smaller particles to larger particles, decreases with higher laser power: from 7.9 to 2.4, with the largest drop between 150 and 200 mW. Those results are summarised in Table 1.

**Table 1:** Diameter of fabricated NPs with the position of absorption peak

Power *P* (mW)	Absorption peak *λ*_max_ (nm)	Smaller particles *d*_<80_ (nm)	Larger particles *d*_>80_ (nm)	Particle ratio *Θ* (1)
100	525	34.0	236	7.9
150	536	37.3	227	6.2
200	547	41.8	201	2.4

### 2.2 Insights from simulations

The observed blueshift of the absorption peak with decreasing particle diameter follows a recognised trend.^48^ However, when we tried to correlate these shifts with numerical simulations, we saw that the change in particle size alone did not adequately explain the observed shift. The maximum absorption wavelength for plasmonic nano-islands also strongly depends on their shape, which is primarily determined by the metal-substrate wetting angle.^49,50^ Thus, we used numerical simulations to obtain further insights into the particle size and shape distribution.

As the basis of our analysis, we calculated a wide range of extinction spectra for particles with diameters between 20 and 600 nm and contact angles between 50 and 170° using finite-element simulations (325 simulations in total) [summarised in Fig. S2.1 in the (SI)]. We then used a least-squares optimisation procedure to fit the calculated spectra to the measured ones, which gives us information about particle size and shape (contact angle) distribution. This approach is challenging because of the limited information available taken from the measured spectra and SEM analysis.

To reduce the number of fitting parameters, we adopted a simplified model assuming two distinct particle populations in the sample, with diameters either below or above 80 nm. For both kinds of particles, we assume a single contact angle and a log-normal size distribution. The least-squares optimisation yields the contact angles and distribution parameters for the two groups of particles. Importantly, we determined the relative populations of the two groups from the fitting, not from SEM images (see S3 in the SI). Furthermore, we ignore the possible resonance shifts due to coupling between the particles.^51^ This is motivated in part by our preliminary simulations, which indicate that the coupling effects are not particularly strong in this case. Additionally, given the already underdetermined nature of the problem, including more degrees of freedom would complicate the data analysis.

**Fig. 3:**
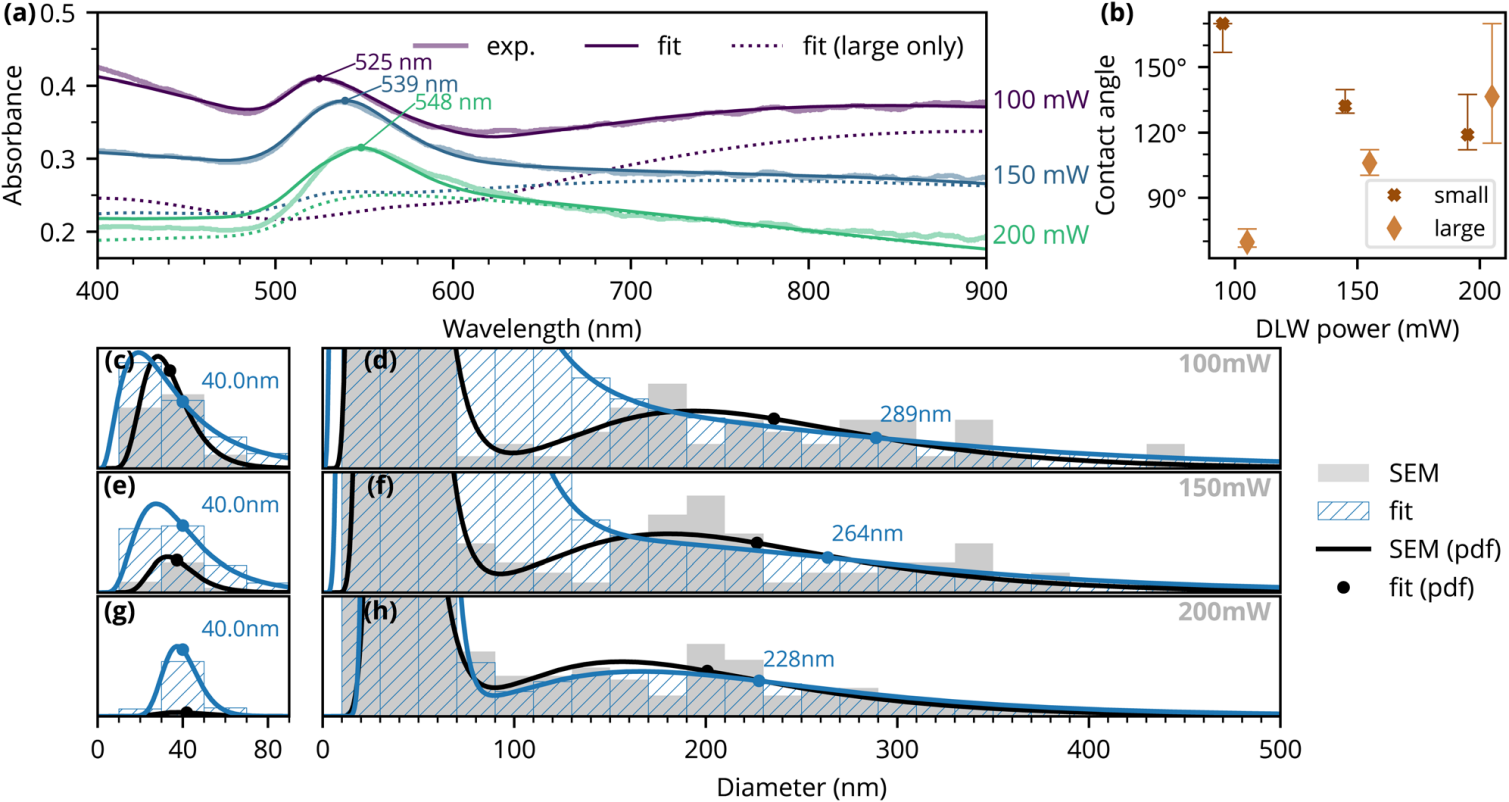
(a) Experimentally measured absorbance spectra (thin light lines), calculated spectra based on least-squares fits using particle scattering cross-sections from full-wave simulations (thin dark lines) and the calculated spectra without contributions from the smallest (*r* = 10 nm) particles. Colors indicate different samples. (b) Wetting angles resulting from fits under assumption of separate constant wetting angle for small and large particles. (c–h) Histograms of particle diameter as obtained from SEM (in gray) and *via* the least-squares fitting (in blue). Corresponding log-normal probability density functions (pdf) are indicated with lines and the mean values are labeled with dots.

The resulting fits from our analysis are shown in Fig. 3(a). Overall, the fitted spectra match the experimental data sufficiently well. Comparing the least-squares results under various assumptions led us to three key insights: (1) the observed absorbance peak is mostly related to plasmonic resonances in small particles, and (2) the particle shapes must vary between the three analysed samples, since size distribution differences alone are not sufficient to explain the changes observed in the spectra, and (3) the contact angles of particles must vary between the small and large particles on each sample.

Firstly, comparing the fitted spectra in Fig. 3(a) with the contribution from the large particles (dotted lines in the figure), we see that the observed absorbance peaks are, for the most part, determined by the small particles. The absorbance peaks correlate directly with the plasmonic resonances of the small particles and, consequently, with their contact angle. This indicates that the retrieved trends for small particles should be robust with regard to the model's simplifying assumptions.

Secondly, the analysis shows that the change in the size distribution of the particles alone is not enough to explain the differences in measured spectra between the three samples. Thus, the particle contact angle must change with the DLW power and the experimentally observed shifting of the peak (with increasing DLW power) is likely due to changes in the contact angle of the small particles. Furthermore, even when different wetting angles are assumed for each sample (identified by laser power), there remains a large mismatch between the fitted spectra and the experimental data (Fig. S4.1 in the SI). Thus, we also need to assume different contact angles for the small and large particles within one sample.

Interestingly, we see that the trends for the wetting angles are opposite for these two kinds (= size fractions) of particles [see Fig. 3(b)]. The trend towards smaller wetting angles in smaller particles is clearly linked to the observed absorbance peak, as explained above. However, smaller particles do not significantly contribute to the measured absorbance above 600–700 nm. So, within our assumptions, the changing contact angle of larger particles is linked to the slope of the absorbance tail. For the 100 mW sample, we see a next peak appearing at the red end, but at higher DLW powers, the higher resonances are shifted further away. In our calculations, both an increase in the contact angle and a decrease in the mean particle size contribute to this shift.

We also show error bars in Fig. 3(b) for the estimated contact angles. The error bars are based on running the fitting procedure with slightly varying optimisation objectives, obtaining slightly different solutions (see Fig. S5.1 in SI). Importantly, we see large variations for the 200 mW sample, which is also the sample with the largest least-squares residual. This result indicates that our assumptions might be too restrictive; for example, there could be more variations in the particle shapes, or the interparticle coupling might become more important here. The latter possibility is supported by reduced interparticle spacing observed in the 200 mW sample (see Fig. S6.1 in the SI).

Fig. 3(c–h) present the particle size distributions obtained through least-squares fitting. The plots show the sum of the two log-normal distributions (for smaller and larger particles). These distributions, which consider both SEM image data and computed absorbance spectra, differ slightly from those of Fig. 2. The results consistently indicate a decrease in the mean diameter of larger particles with increasing laser power, from approximately 290 nm to 230 nm.

For small particles, we fixed the mean diameter at 40 nm because the effects of varying the particle size and concentration are nearly indistinguishable, leading to a large variance in the determination of both parameters during the fitting procedure. To simplify the analysis, we fixed one of these parameters. This mainly affects the fitted population counts; the trends observed in the retrieved contact angles remain. The same is not necessary for larger particles, as in their case, the changes in particle size significantly alter the spectral shape of absorbance.

### 2.3 Thermal annealing: investigation of melting process

**Fig. 4:**
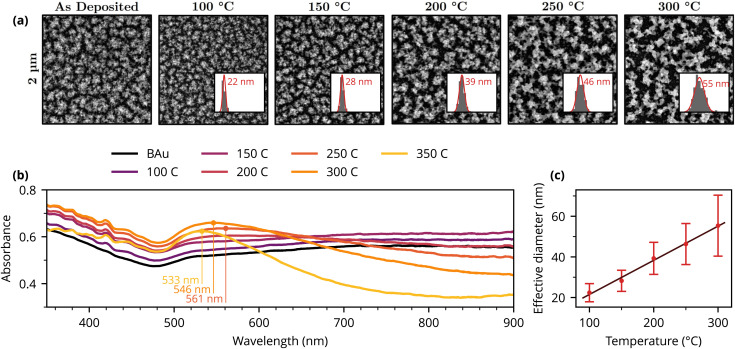
(a) SEM images of the thermally annealed BAu structures, inset show distribution of equivalent particle diameters (with mean indicated). (b) Absorbance spectra of untreated and thermally annealed BAu structures. (c) Dependency of equivalent diameter on annealing temperature.

To investigate the melting process that was first observed during the black metal deposition and subsequently led to the idea to use direct laser writing (DLW) for the fabrication of plasmonic NPs on BAu films, thermal annealing of nanostructured BAu layers was performed. Fig. 4(a) shows the SEM images of as-deposited and subsequently thermally annealed BAu layers. The morphology of as-deposited film corresponds to those prepared and investigated earlier^17,18,52^ showcasing the typical highly nanoporous and nanostructured cauliflower-like or spongy-like surface morphology. The images illustrate the sintering and recrystallisation of the nanostructured black gold layer. The particle analysis of nanostructured clusters done on SEM images proved that the provided thermal energy causes the collapse and meltdown of the nanostructured clusters of black gold, resulting in larger particles. This sintering effect was originally observed also by Harris *et al.*,^5^ who identified the 100 °C as the temperature above which the sintering process occurs rapidly, when investigating the change in electrical resistance of the black gold film with temperature.

The insets showing the particle distribution in individual SEM images illustrate the increase in the mean equivalent diameter. As illustrated in Fig. 4(c), this pattern exhibits a linear correlation with the annealing temperature and provides an efficient tool for tailoring the average particle size. Consequently, therefore, it is possible to manage the average particle size by adjusting the annealing temperature.

The absorbance spectra of the as-deposited and annealed BAu films measured in the visible region are depicted in Fig. 4(b).

Compared to the DLW samples (Fig. 1) the thermally annealed films show a more broadband response.

The spectra were measured at three different spots with only a slight difference in overall intensity, demonstrating the homogeneity of the treated areas. The meltdown of the clusters is shown to induce a decrease in absorbance in the region of 650–900 nm starting with the annealing temperature of 200 °C.

Higher annealing temperatures, starting from 250 °C, caused the appearance of an absorption peak with a maximum around 550 nm. This peak then shifts towards lower wavelengths with higher annealing temperatures: specifically 557 nm for 250 °C; 547 nm for 300 °C; and 534 nm for 350 °C. This observation goes a little against the usual trend of plasmonic behaviour of gold nanoparticles, where the absorption peaks shift towards shorter wavelengths with a decrease in particle size,^53^ but on the other hand, as is clear from the SEM images [Fig. 4(a)], those are not typical plasmonic nanoparticles, but rather highly porous nanostructures of gold. Here the optical absorption is strongly influenced by multiple scattering/light trapping and plasmonic excitations associated with voids and irregular nanostructures rather than a single-particle dipolar LSPR alone.^10^

The thermally annealed samples show little difference in the absorbance spectrum below 500 nm across different annealing temperatures, unlike the DLW-treated samples. This suggests that the response might be driven predominantly by light trapping and ohmic losses in the metal.^16^ However, as revealed recently, broadband localised plasmon resonances indeed play a role in the optical properties of nanoporous gold layers.^54^ We thus believe that the optical properties of evaporated black gold result from both effects of broadband LSPR and multiple scattering/light trapping. Notably, the sample annealed at 350 °C begins to show bigger changes in the spectral shape, indicating an increasing role of more particle-like resonances, as seen in the DLW-treated samples.

## 3 Conclusions

We have presented the study dedicated to modifying of black gold samples by two techniques—direct laser writing or heat treatment—after which the samples exhibit plasmonic resonances. In both cases, the resonance wavelength can be tuned by the treatment parameters. Using SEM imaging and numerical analysis, we connect, in DLW-treated samples, the observed shift of absorbance spectra to shape changes of the formed plasmonic nanoparticles. The study revealed the formation of two distinct particle populations, large-sized and small-sized, in the DLW samples.

Varying the DLW laser power resulted in contrasting size trends for these particles: large particles exhibited decreasing diameters, while small particles showed a slight increase in size with increasing laser power. Numerical analysis further indicated that these particle populations form with different contact angles and respond differently to the increase in DLW power.

By contrasting the two treatment processes, we highlight the different underlying mechanisms for light absorption in the black gold sample (localized plasmonic resonances *vs.* broadband light trapping). The plasmonic absorbance peak appears in both experiments, but the direction of the spectral shift under treatment strength differs.

Formation of the nanoparticles in the DLW-treated samples is controlled by (1) the initial BAu thickness and morphology (determined by the deposition parameters) and (2) the delivered photothermal dose (*i.e.*, laser power and scan speed). Together, these parameters determine the extent of local reflow/coalescence and, in turn, the resulting particle size distribution and LSPR response. After the sample treatment and formation of the plasmonic particles, subsequent selective laser treatment could be applied by exploiting different absorption cross sections of the two particle populations. This provides a comparatively accessible route to mask-free, patterned nanoparticle formation, which may be particularly attractive for applications requiring spatially selective and delicate patterning of plasmonic features. Moreover, our findings motivate future studies to investigate the particle formation process in more detail. More detailed characterization methods would allow extending the model to account for more complex interactions, such as possible plasmonic coupling between the particles. Numerical simulations of laser heating and subsequent particle formation would help to understand the mechanism behind the observed trends.

## 4 Experimental

### 4.1 Sample preparation

#### 4.1.1 Fabrication of black gold layers

The black gold (BAu) layers were fabricated by a thermal evaporation method in an inert atmosphere of argon gas (Ar) using a tungsten boat. The Zahoxin KXN-15200D DC power supply was used as a current source. The glass and silicon substrate samples were mounted on a stainless-steel holder at a distance of 60 mm above the tungsten boat. A pure gold pellet was used as the source material for the evaporation. The deposition was carried out in five steps with a heating current of 100–130 A. It was experimentally verified that the heat radiation from the tungsten boat can heat the samples to the temperature where the nanostructured surface of black gold starts to melt down. To prevent his undesirable effect, the substrate temperature was measured, and the evaporation was stopped before reaching a temperature of 50 °C and all samples used in this manuscript were prepared within the same deposition batch to avoid batch-to-batch variability. The deposition conditions are summarised in Table 2. The observed deposition conditions are shown in Fig. S8.1 in the SI.

**Table 2:** Deposition conditions

Quantity	Value
Base pressure	5.5 × 10^−4^ Pa
Working pressure	100 Pa
Heat power	<340 W
Substrate temperature	<50 °C

#### 4.1.2 Thermal annealing of black gold layers

To investigate the melting process of the nanoparticles, the prepared black gold (BAu) layers were subsequently treated by thermal annealing using a mini hotplate MiniWare MHP30M. The layers were heated in an ambient atmosphere for ten minutes at 100, 150, 200, 250, 300 °C.

#### 4.1.3 Direct laser writing (DLW) of black gold layers

Plasmonic nanoparticles (NPs) were fabricated by direct laser writing of black gold using a laser deposition system consisting of a continuous-wave laser diode operating at 405 nm, and a 4× microscope objective attached on the XYZ positioning stage. The energy density of the laser radiation incident per area unit was controlled by altering the laser scan speed in the range of 0.012 to 10 mm s^−1^ and controlling the power source from 20 to 200 mW. The laser beam was focused on the sample using Dinoeye AM4023 (Dino-Lite) and an optical path consisting of laser filters and a mirror. The laser spot size focused on the sample plane was approximately 5.6–8.8 m (see Fig. S9.1 and Table S1).

### 4.2 Morphology characterization

#### 4.2.1 Scanning electron microscopy (SEM)

The surface morphology of the prepared black gold films before and after direct laser writing was characterised by scanning electron microscopy (SEM) using the Mira 3 Tescan field emission electron microscope at high magnifications (50k× and 100k×) with a perpendicular in-beam secondary electron detector at an accelerating voltage of 10 kV. The layer thickness was determined by sample cross-sectional measurement starting from the cut of glass and silicon substrates. Particle size analysis was performed from SEM images using ImageJ software.

#### 4.2.2 Atomic force microscopy (AFM)

Atomic force microscopy Bruker Multimode 8 equipped with Nanoscope V electronics was performed to investigate the morphology and roughness of the surface. Measurements were made under ambient conditions and images were obtained by Peak Force Tapping mode with scan areas of 2 × 2 µm^2^.

### 4.3 Investigation of optical properties

#### 4.3.1 UV-vis spectroscopy

The absorbance spectra of the prepared and laser treated black gold samples were measured using a set-up consisting of a light source DH-2000-BAL Ocean Insight and a spectrometer HR2000+ ES Ocean Insight. The film deposited on the glass substrate was illuminated with a circular spot and measured in transmission arrangement. The diameter of all fibres was 450 µm. The reflectance spectra were acquired in the spectral range of 350 to 900 nm. We estimate a measurement error of less than 2% in the UV region and less than 1% in the visible region.

### 4.4 Finite element simulations

For numerical simulations, we use the finite-element software package COMSOL Multiphysics. We simulate individual gold particles on a glass substrate (*n* = 1.5233) under a normally incident plane wave illumination. For the refractive index of gold, we use data from Johnson and Christy.^55^ From the simulations, we obtain cross sections for absorption, forward scattering (*i.e.*, into the substrate) and backward scattering.

### 4.5 Least-squares fitting

The fitting procedure optimized eight parameters: a parameter *l* accounting for scattering losses, and for each of two particle species, a contact angle, a relative weight, and lognormal size distribution parameters (scale *s* and shape *σ*). For the smaller particle species, the mean diameter was fixed to 40 nm, reducing its distribution to one free parameter. The parameter l represents the fraction of forward-scattered light that does not reach the detector, with *l* = 0 corresponding to complete collection and *l* = 1 to total loss.

Optimization was performed by minimizing a weighted objective function, which included terms for the mean-squared error (MSE) of the spectra and the log-likelihood of the particle size distribution. To ensure a robust search of the parameter space, 2500 independent fits with randomized initial conditions and objective weights were run for each sample. Unsuitable solutions were subsequently filtered based on criteria including the spectral MSE, distribution likelihood, and the position of the absorbance peak. The complete fitting procedure and code to reproduce Fig. 3, S3.1, S4.1 and S5.1 are available at https://github.com/taavirepan/bau_fitting.

## Author contributions

Taavi Repän: methodology, investigation, conceptualization, writing – original draft. Martin Hruška: methodology, investigation, conceptualization, writing – original draft, visualization. Jaroslav Otta: methodology, investigation, conceptualization. Přemysl Fitl: supervision, validation, funding acquisition, project administration. Michal Novotný: supervision, validation, funding acquisition, project administration. Tereza Hodná: investigation Martin Vrňata: conceptualization, supervision, funding acquisition, project administration, revision. Raivo Jaaniso: supervision, funding acquisition, project administration, revision.

## Conflicts of interest

There are no conflicts to declare.

## Supplementary Material

RA-016-D6RA00590J-s001

## Data Availability

The data and code to reproduce the numerical analysis is available at https://github.com/taavirepan/bau_fitting (https://doi.org/10.5281/zenodo.20343395). Supplementary information (SI) is available. See DOI: https://doi.org/10.1039/d6ra00590j.
