# Supplementary material for: Direct laser writing of black metals for tuneable plasmonic nanoparticles: experimental and computational insights
Source: RSC Adv. 2026 May 27;16(31):28526–34. doi: 10.1039/d6ra00590j (PMC13217168; doi:10.1039/d6ra00590j)
Supplement: RA-016-D6RA00590J-s001 [file RA-016-D6RA00590J-s001.pdf]

Supplementary Information (SI) for RSC Advances.
This journal is © The Royal Society of Chemistry 2026

Direct Laser Writing of Black Metals for Tuneable Plasmonic Nanoparticles: Experimental and Computational Insights

Supplementary Information

S1 AFM images

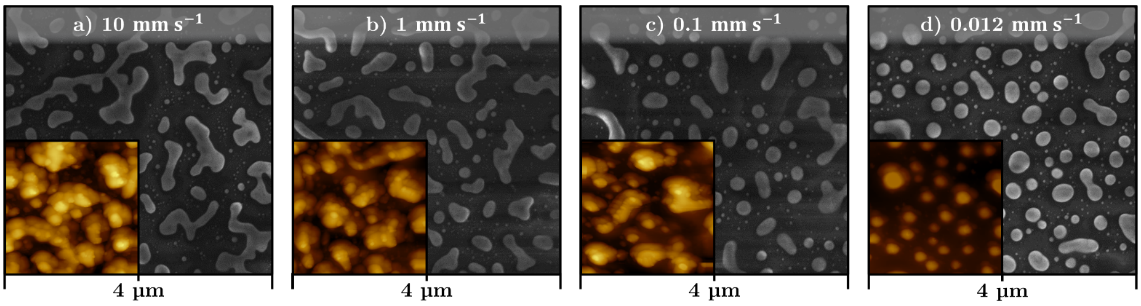

Figure S1.1: SEM and AFM images illustrating the formation of plasmonic nanoparticles using different DLW scan speed with fixed power of 50 mW.

S2 Dataset of particle extinction spectra

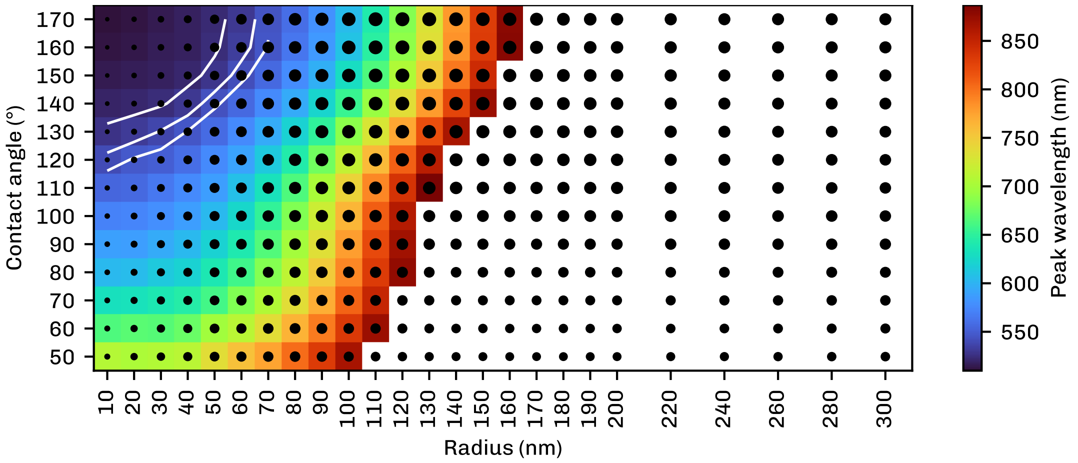

Figure S2.1: Summary of the generated dataset of particles. Color scale indicates location of the first extinction peak. For uncolored samples the first peak was outside of the simulated wavelength range. Black dots indicate relative extinction efficiencies. The white lines indicate experimentally observed peak locations (525, 537 and 546 nm).

Figure S2.1 summarizes the generated dataset of extinction spectra of different particles. Particles themselves are illustrated in fig. S2.2. Each square in the figure corresponds to one simulation, done for a specific particle radius and contact angle. Each simulation contains wavelengths from 400 to 890 nm[1], in 10 nm increments. Between r=10 nm and r=200 nm, the simulations were done with 10 nm increment for the radius. For larger particles the step was increased to 20 nm, because at those sizes the calculated spectra were less sensitive to the particle size. Larger stepping helped to reduce computational costs.

[1]900 nm is missing from the sweep due to particular setup of the wavelength sweep, where the upper bound was not included in the sweep.

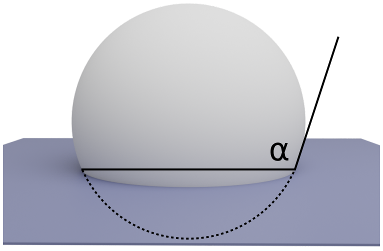

Figure S2.2: Illustration of the simulation geometry. An individual (hemi)spherical particle is simulated on a glass substrate. The shape is parametrized by contact angle α and radius.

S3 Ratio of small to large particles

As discussed in the manuscript, we have obtained particle size distribution information from SEM images. We have chosen d=80 nm as the cutoff between the small and large particles. Based on the measured distributions we can then estimate the ratio of number of small particles compared to the large ones (shown in the table below).

However, as fig. S3.1 shows, using the measured ratios yields worse fits compared to leaving the particle ratio as one of the degrees of freedom in the least-squares minimization. Given limited SEM resolution and possible issues with charging of the sample it might be that smallest particles are underrepresented in distributions obtained from the image analysis. Thus it makes sense to leave the ratio of small particles as additional degree of freedom.

S4 Fitted spectra, assuming uniform contact angles

Figure S4.1(a) shows fitted spectra under different assumptions for the relationship between the contact angle vs the particle radius. Firstly, considering the assumption of uniform contact angles (the red lines in the figure), we note that the resulting fits are significantly worse than those used in the main manuscript (blue lines in the figure), especially for the 100 mW sample.

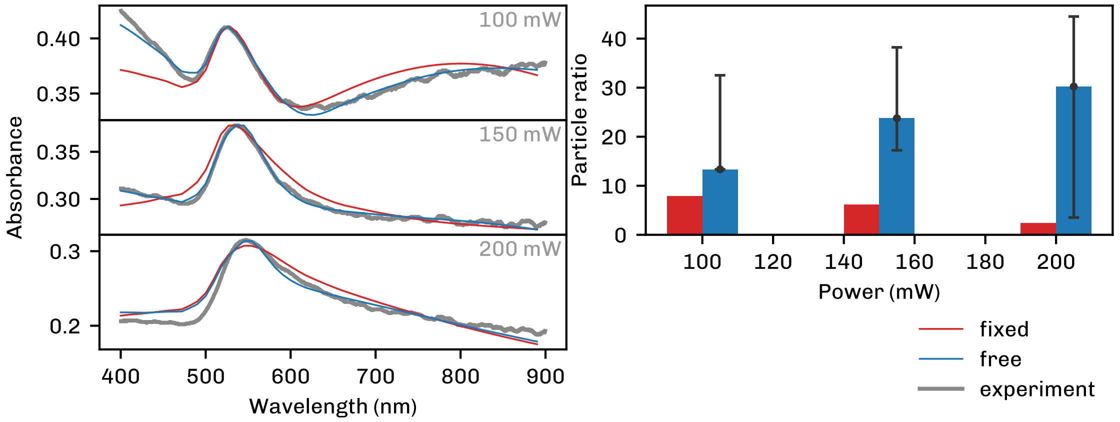

Figure S3.1: (**a**) Comparison of the results shown in the main manuscript (blue) and results where ratio of small and large particles was fixed to data from the SEM. (**b**) Ratio of small to large particles, either from measurement (red) or least-squares fitting (blue). Errorbars are based on the results shown in section S5.

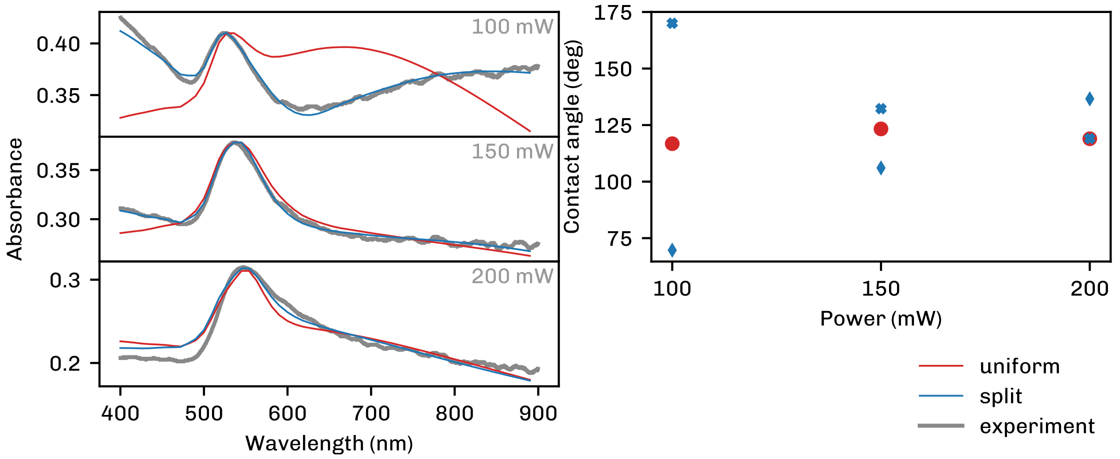

Figure S4.1: (**a**) Fitting results under different models for the contact angle. The red lines represent the assumption of uniform contact angles for both small and large particles. The blue lines show the results presented in the main text, where small and large particles were fitted with separate contact angles. (**b**) Resulting contact angles, with red markers indicating the uniform assumption and blue markers showing the main results. Blue diamonds indicate large particles and crosses indicate small particles.

S5 Variability of results

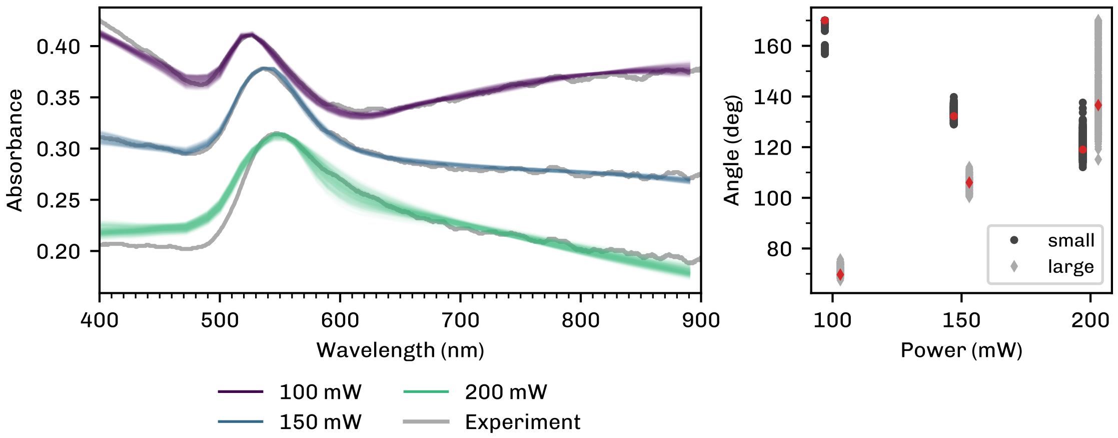

Figure S5.1: Overview of fitting results after filtering showing plausible results based on different random weights for the optimization objectives. Red markers show the best fit.

S6 Particle separation distribution

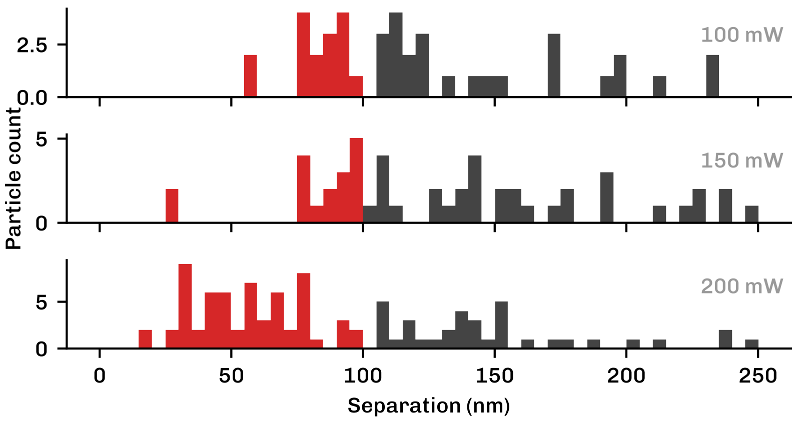

Figure S6.1: Histograms of particle separations for the three samples, based on data extracted from SEM images, only including particles with diameter at least 100 nm. The red area indicates separations up to 100 nm, which is the range where simulations suggest increasing importance of inter-particle coupling.

S7 Stability of the samples

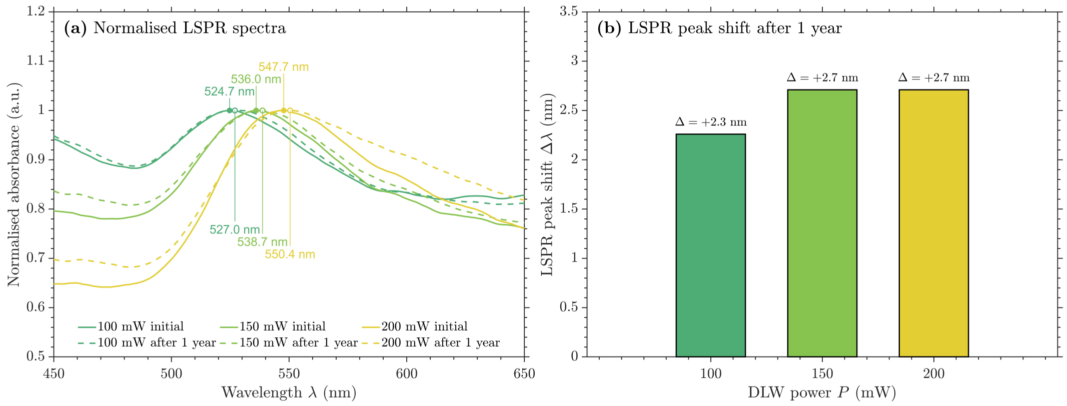

Figure S7.1: **a**) Absorbance spectra measured one year after fabrication for DLW-treated samples (100, 150, and 200 mW), compared with the original spectra. **b**) Change in LSPR peak position after one year.

S8 Deposition conditions

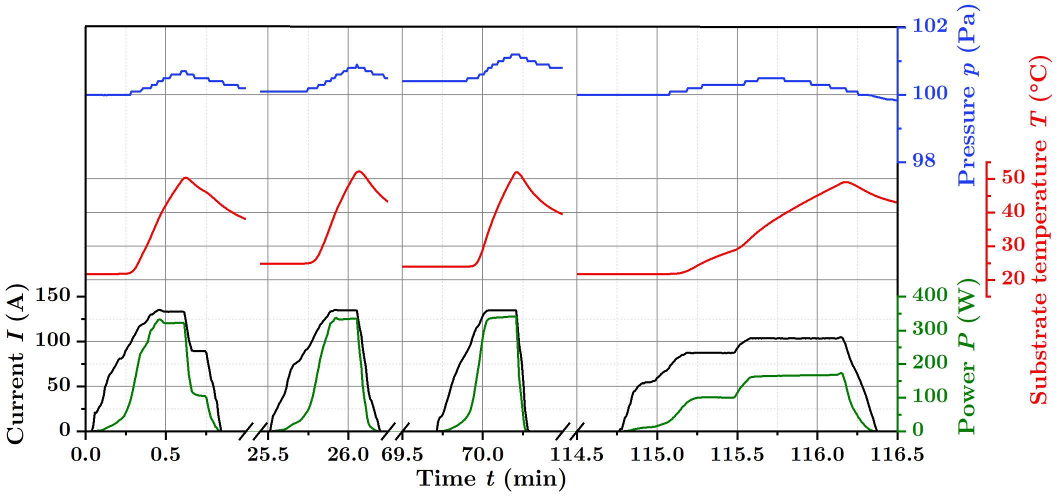

Figure S8.1: Deposition conditions during stepwise thermal evaporation of BAu. The deposition is split into short evaporation steps with cooling pauses to prevent radiative heating from exceeding the 50 °C limit.

S9 DLW – Laser parameters

Table S1: Effective focused spot diameter and corresponding average irradiance assuming a circular footprint ($I_{avg} = P/(\pi(d/2)^2)$).

d (µm)	I_{avg} at 50 mW (kW cm^{-2})	I_{avg} at 200 mW (kW cm^{-2})
5.604	203	811
8.793	82	329

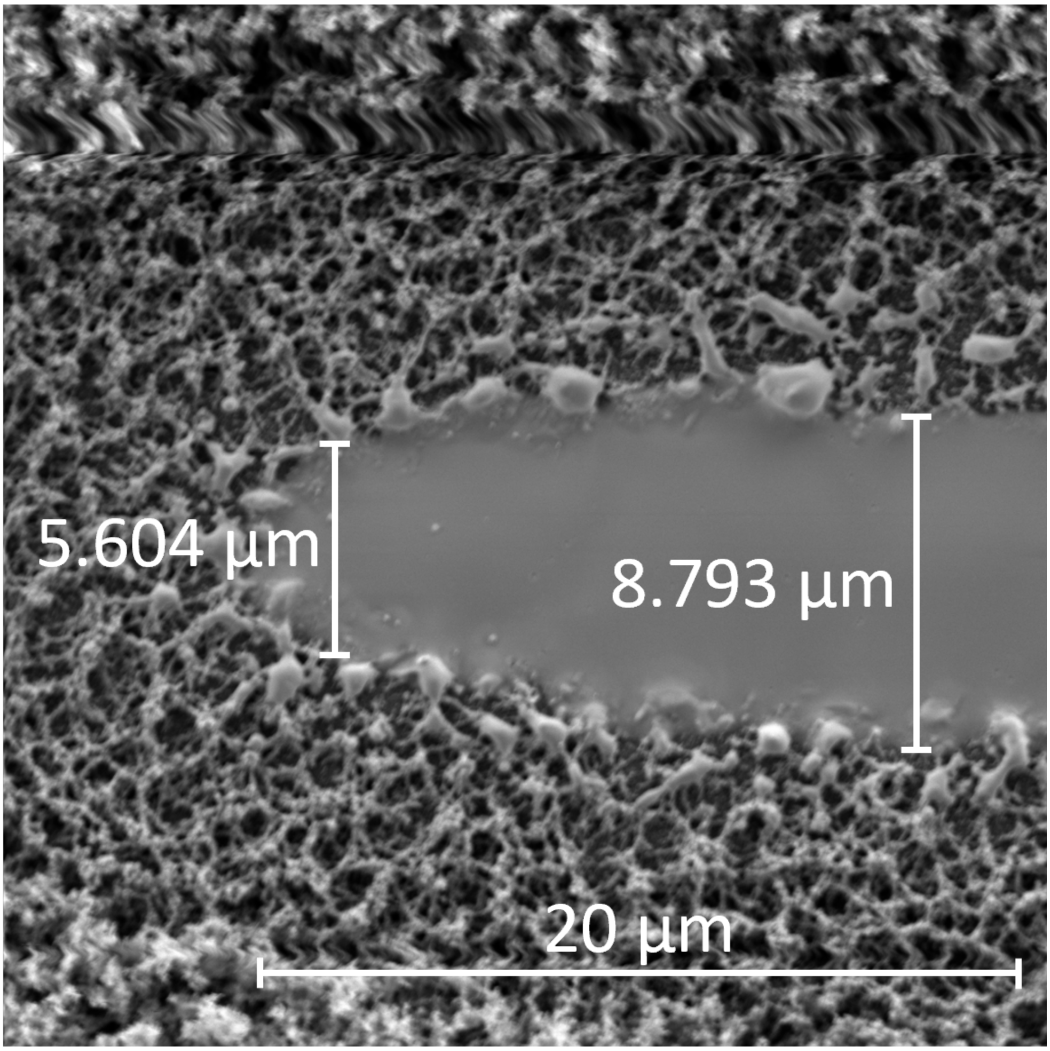

Figure S9.1: Estimated focused laser footprint at the sample plane from the laser-modified region on a black bismuth reference film, yielding an effective spot diameter range of 5.604–8.793 µm.
